# Saffron (*Crocus sativus* L.) in Ocular Diseases: A Narrative Review of the Existing Evidence from Clinical Studies

**DOI:** 10.3390/nu11030649

**Published:** 2019-03-18

**Authors:** Rebekka Heitmar, James Brown, Ioannis Kyrou

**Affiliations:** 1 Aston University, School of Life and Health Sciences, Aston Triangle, Birmingham B4 7ET, UK; j.e.p.brown@aston.ac.uk; 2 Aston Medical Research Institute, Aston Medical School, Aston University, Birmingham B4 7ET, UK; I.Kyrou@aston.ac.uk; 3 WISDEM, University Hospitals Coventry and Warwickshire NHS Trust, Coventry CV2 2DX, UK; 4 Translational & Experimental Medicine, Division of Biomedical Sciences, Warwick Medical School, University of Warwick, Coventry CV4 7AL, UK

**Keywords:** saffron, *Crocus Sativus* L., crocin, crocetin, supplements, anti-oxidant, anti-inflammatory, AMD, diabetes, glaucoma

## Abstract

Saffron (*Crocus sativus* L.) and its main constituents, i.e., crocin and crocetin, are natural carotenoid compounds, which have been reported to possess a wide spectrum of properties and induce pleiotropic anti-inflammatory, anti-oxidative, and neuroprotective effects. An increasing number of experimental, animal, and human studies have investigated the effects and mechanistic pathways of these compounds in order to assess their potential therapeutic use in ocular diseases (e.g., in age related macular degeneration, glaucoma, and diabetic maculopathy). This narrative review presents the key findings of published clinical studies that examined the effects of saffron and/or its constituents in the context of ocular disease, as well as an overview of the proposed underlying mechanisms mediating these effects.

## 1. Introduction

In addition to uncorrected refractive error, age related macular degeneration (AMD), glaucoma, cataract, and other retinal diseases (e.g., diabetic retinopathy and retinitis pigmentosa) are the major causes of blindness worldwide [1,2,3,4]. Among these ocular diseases, AMD is currently listed as the leading cause of irreversible vision loss in the developed world [2]. While the introduction of anti-VEGF (vascular endothelial growth factor) treatment has had a positive impact on preserving vision and slowing progression in AMD [5], there is still no cure to date. Furthermore, the increasing prevalence rates of obesity and related cardio-metabolic disease, including type 2 diabetes mellitus (T2DM) and cardiovascular disease (CVD) [6], adds significantly to the imposed health care burden and increases the treatment challenges. Indeed, increasing data link AMD to a number of lipid pathway genes, CVD phenotypes, excess body weight, and central/abdominal obesity [7,8,9]. Moreover, most of the aforementioned conditions are also associated with aging and exhibit overlapping pathophysiology with common mechanistic pathways, such as inflammation, oxidative stress, apoptosis, and neurodegeneration [6,7,10,11,12,13]. As these pathways may be affected by the effects of various nutritional supplements and botanical/herbal compounds (e.g., saffron and its constituents), increasing research interest is now focused on the potential therapeutic use of such natural products [11,14,15,16]. 

Saffron is mainly used in cooking as a colouring and flavouring spice that is comprised of the dried stigmas of the *Crocus sativus* L. flower, a stemless herb that belongs to the Iridaceae family [17,18]. Based on phytochemical studies, the pharmacologically active saffron constituents include bitter principles (e.g., picrocrocin), volatile agents (e.g., safranal, which can be obtained by picrocrocin hydrolysis), and dye materials (e.g., crocetin and its glycoside, i.e., crocin, which gives saffron its characteristic colour) [17,18,19]. In herbal medicine, saffron is traditionally used as a nerve sedative, stress-reliever, anti-depressant, aphrodisiac, expectorant, and anti-spasmodic agent [17,18,20,21]. Growing evidence from pharmacological studies has further shown that saffron or its active constituents may potentially exert neuroprotective, anti-convulsant, anti-depressive, anxiolytic, anti-oxidant, anti-inflammatory, hypolipidemic, anti-atherogenic, anti-hypertensive, and even anti-tumour effects [17,18,20,22,23]. Of note, a recent meta-analysis by Pourmasoumi et al. showed that saffron may be beneficial for several CVD-risk related outcomes (e.g., blood pressure, body weight, waist circumference, and fasting blood glucose levels), suggesting that saffron may have protective effects for multiple systemic conditions related to such CVD risk factors [24]. Furthermore, favourable results have been recently reported from both animal and clinical studies examining the effects of saffron and its constituents on CVD risk [22,24,25], endothelial function [26], inflammatory diseases [23,27], oxidative stress [26,28], and glycaemic factors [24,29], indicating that saffron may have promising potential as adjunct therapy in both systemic conditions and ocular diseases mainly through its anti-inflammatory and anti-oxidative effects [14,17,20,22]. 

This narrative review presents key findings of clinical studies that investigated the impact of saffron or one of its constituents on vision-related outcome measures, and an overview of the proposed mechanisms mediating these effects. As saffron is a natural product and, hence, saffron supplements are under less tight regulation regarding sale and dosage, this review is also intended to provide concise information on dosage and potential side effects based on the available literature.

## 2. Methods

Although not a systematic review, in order to ensure the quality and consistency of our approach, in the present narrative review we applied a predefined search strategy and followed, where relevant, the Preferred Reporting Items for Systematic Reviews (PRISMA) guidelines [30,31].

*Search strategy*: A systematic search of the following databases was conducted: PubMed, Scopus, Google Scholar, Cochrane library, and Web of Science. Studies published on these databases available up to the 31st of January 2019 were included for screening. Using Boolean operators (e.g., AND, OR), the applied search terms included combinations of the following key words: “saffron”, “safranal”, “crocetin”, “crocin”, “eye”, “ocular”, “retina”, “diabetes”, “macula”, “glaucoma”, “age related macular degeneration”, “AMD” “anti-inflammatory”, “anti-oxidant”, “neuroprotective”, “nutrition”, and “supplement”. Only publications in the English language were included. To minimise the risk of omitting relevant studies, the reference lists of all eligible papers were also manually checked. 

*Study selection*: After elimination of duplicate records by one author (RH), two authors (RH and IK) independently reviewed the remaining publications to decide which were suitable for inclusion in this review. As a first step to eliminate unsuitable studies, all titles and abstracts of the publications identified through the performed database searches were screened, and studies that were clearly irrelevant were removed. Subsequently, the remaining papers were evaluated by reviewing the full text versions. Finally, all clinical studies in adults with ocular diseases which assessed the impact of saffron and/or its constituents on vision-related outcome measures, such as visual acuity, visual field parameters, contrast sensitivity, electrophysiology parameters (electroretinography (ERG), focal ERG (fERG), and multifocal ERG (mfERG)), macular thickness measures, and intraocular pressure (IOP), were included and reviewed in detail. Any discrepancies regarding inclusion of clinical studies were resolved by consensus and, if necessary, discussion with a third author (JB). Table 1 presents the PICOS (Population/Participants/Problem, Intervention, Comparators, Outcomes, Study Design) criteria that were followed in order to identify clinical studies for the present review.

## 3. Clinical Evidence Regarding the Impact of Oral Supplementation with Saffron or One of Its Constituents on Vision-Related Parameters in Adults with Ocular Diseases

Based on the results of the performed literature searches, there were eight published clinical studies that have assessed the impact of oral supplementation with saffron or one of its constituents on vision-related parameters in adults with ocular diseases [32,33,34,35,36,37,38,39]. Of these, six were randomized controlled trials (RCTs) [32,35,36,37,38,39], while two are longitudinal interventional clinical studies reporting on pre- (baseline) versus post-intervention comparisons without a comparator/control group [33,34]. For the two latter studies that are from the same group and report on different outcome measures, it is also not clear whether their design involves, at least partly, participants of the same cohort, and efforts to contact the corresponding authors in order to clarify this point were not successful [33,34]. The key characteristics and findings of these eight clinical studies are presented in the following sections and are summarized in Table 2.

*Age related macular degeneration*: six published clinical studies assessed vision-related parameters in AMD patients under oral saffron supplementation (Table 2) [32,33,34,35,36,37]. Based on these, both objective (ERG) and subjective measures (Snellen, LogMar, EDTRS charts) of visual acuity were shown to significantly improve with all tested dosages of saffron (daily dose range: 20–50 mg), even after short-term oral supplementation (e.g., three months) [32,33,34,35,36,37]. Central macular thickness (CMT) [35] and contrast sensitivity (CS) [36] were also assessed in certain studies (Table 2), with the latter reportedly increasing with saffron supplementation [36], whilst the former was shown to decrease only in wet, but not dry AMD following saffron supplementation [35]. As the formulation, dosage, intervention duration, test methods, and outcome measures varied across these six studies, a direct quantitative comparison was not possible. Of note, longer-term data are currently available only from the two non-RCT studies with oral saffron supplementation (20 mg daily) in patients with bilateral early AMD over 12 and 15 months, respectively (Table 2) [33,34]. In these two longer studies, the noted improvements were achieved within three months of oral saffron supplementation, after which the gained functionality seemed to plateau [33,34]. 

*Glaucoma*: the only existing clinical study in patients with primary open angle glaucoma (POAG) reported that oral saffron supplementation (30 mg daily for one month) can significantly reduce the IOP after three weeks (Table 2) [38]. In this pilot double-blind, placebo controlled RCT, all participants had stable POAG (verified by visual field and optic nerve head examinations) and were treated with topical timolol 0.5% twice daily and dorzolamide 2% three times daily. Compared to placebo, the addition of daily oral saffron supplementation for one month resulted in significantly decreased IOP after the third week, but this effect reverted after a 4-week wash-out period [38].

*Diabetic maculopathy*: despite the alarmingly increasing T2DM prevalence, only one eligible RCT was identified in patients with refractory diabetic maculopathy; it examined the effects of oral crocin supplementation (5 mg or 15 mg daily) (Table 2) [39]. In this study, Sepahi et al. reported a decrease in CMT that appeared dose dependent, showing significant reduction after three months of daily oral crocin supplementation compared to placebo in the higher (15 mg), but not in the lower (5 mg) dosage group [39]. Visual function, as measured by best corrected visual acuity (BCVA), was also significantly improved only in the higher dosage group compared to placebo. Moreover, in addition to the ocular findings, oral crocin supplementation significantly reduced HbA1c and fasting blood glucose levels only with the higher dose. Interestingly, while the lower crocin dose did not induce significant reductions compared to placebo, the study authors noted that this dose could clinically improve CMT, BCVA, HbA1c, and fasting glycaemia [39].

## 4. Action Time-Course of Oral Saffron Supplementation

To date, there are limited data regarding the time-course of the effects of oral saffron supplementation on visual-related parameters in patients with ocular diseases. From most of the aforementioned published clinical studies (Table 2) [32,33,34,35,36,37,38,39], it appears that saturation of the saffron-induced effect(s) is reached within a three-month period of oral supplementation. It is plausible that this saturation can be reached earlier; however, this remains uncertain, since most of these studies included testing at three-month intervals. Moreover, it is noteworthy that in a study utilizing a light damage model of photoreceptor degeneration in rats, the protection noted with the applied daily dose of saffron (1 mg/kg) was detectable at two days, increasing to 10 days [40].

## 5. Safety Profile of Oral Saffron Supplementation

A relatively limited number of studies have examined the toxicity of saffron [18,22,41,42,43]. According to *in vivo* studies, saffron has very low toxicity for doses of up to 1.5 gr per day, while toxic effects have been documented for daily doses ≥5 gr, with a lethal dose of approximately 20 gr [44]. The side effects of saffron are partly attributed to its dye/coloured constituents, which can accumulate in the skin, mucosas, and/or sclera mimicking icteric symptoms [44]. 

Taking into account that toxic effects of saffron appear to require doses of grams per day, clinical data from studies in healthy volunteers suggest that saffron and crocin in doses of milligrams per day can be considered safe [45,46]. Indeed, a randomized, double-blind, placebo-controlled study examining the safety of oral crocin supplementation (20 mg daily compared to placebo) in healthy volunteers concluded that this was relatively safe within the one-month study period [45]. In this short-term study, there were no major adverse events and no changes in the studied haematological, biochemical, hormonal, and urinary parameters, except for decreasing amylase levels, mixed white blood cells, and partial thromboplastin time after one month [45]. Saffron doses of 200 mg and 400 mg for seven days were also shown to be relatively safe in another double-blind, placebo-controlled study in healthy volunteers, with changes in haematological and biochemical parameters that were not considered clinically important [46]. Notably, in this study, one female participant in each of the two saffron groups exhibited abnormal uterine bleeding [46]. 

Furthermore, systematic review data from RCTs examining the effectiveness of saffron on behavioural and psychological outcomes also support the safety of saffron supplementation, since in all these studies there were no significant differences in adverse events between saffron and placebo groups [47]. Nausea, sedation, appetite fluctuation, and headache were among the most common adverse effects reported in these RCTs, which used saffron daily doses ranging from 20 mg to 400 mg (supplements containing additional compounds with putative synergistic effects were also used in some of these RCTs) [47]. Similarly, a good safety profile has been reported in the available clinical studies in patients with ocular conditions, most of whom had regular follow-up that also included telephone calls and interviews to ensure compliance and monitor possible side effects [32,33,34,35,36,37,38,39]. Indeed, these studies concluded that the applied dose regimens of saffron and crocin were safe without significantly increased safety risk [32,33,34,35,36,37,38,39]. As such, the available clinical evidence supports the safety of oral saffron supplementation; however, longer-term studies are still required in order to comprehensively evaluate the long term safety profile of saffron and its constituents in various conditions. 

Overall, caution is currently advised regarding saffron supplementation in patients with renal insufficiency or bleeding disorders, and in those on anti-coagulation treatment due to potential inhibition of platelet aggregation [21,41]. Finally, saffron supplementation has also potential emmenagogue and abortifacient effects [48]. In fact, saffron has been reportedly used for induction of abortion (doses >10 gr) [41,44], with amounts higher than those used in foods (>5 gr) having uterine stimulant effects; thus, it should be avoided during pregnancy [21].

## 6. Proposed Mechanisms/Pathways Mediating the Effects of Saffron and/or Its Constituents in Ocular Diseases

**Figure 1:**
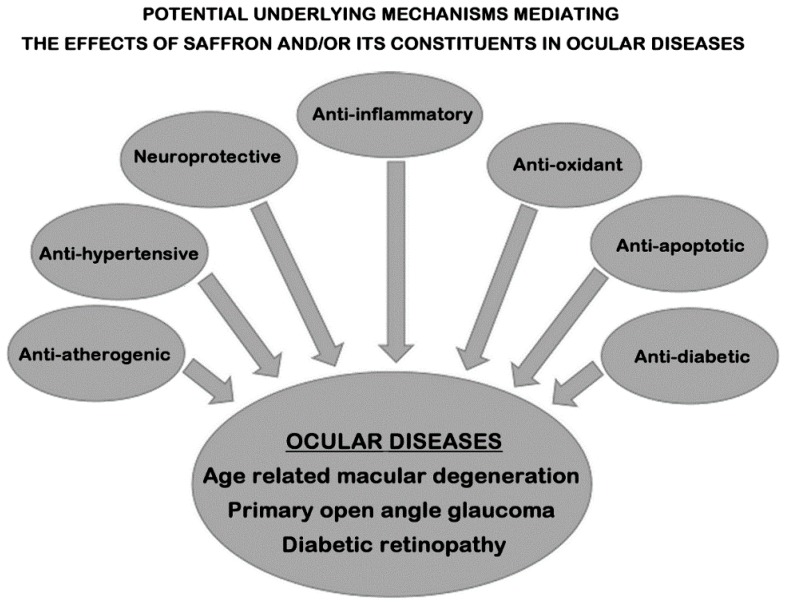
Simplified schematic representation of the potential mechanisms that may mediate the effects of saffron and/or its constituents (e.g., crocin) in various ocular diseases (e.g., age related macular degeneration, primary open angle glaucoma, and diabetic retinopathy), including anti-inflammatory, anti-oxidant, anti-apoptotic, neuroprotective, antidiabetic, anti-atherogenic, and anti-hypertensive effects.

Taking into account the underlying pathophysiology of AMD, diabetic retinopathy, and glaucoma, improvements in the overall pro-inflammatory and oxidative stress load, as well as in the systemic vascular/endothelial health, have the potential to also positively impact on ocular-related parameters. Therefore, given the favourable effects of saffron and/or its constituents on multiple CVD-risk factors and inflammation [23,24,27,28], it is highly plausible that the same underlying anti-oxidant, anti-apoptotic, and anti-inflammatory mechanisms/pathways are also facilitating the noted positive effects of oral saffron supplementation in ocular diseases (Figure 1). For example, reduced circulating levels of glutathione (GSH; a key radical scavenger) have been shown in patients with POAG, leading to increased oxidative stress [49]. Accordingly, a possible mechanistic pathway mediating the effects of saffron and its constituents (crocin and crocetin; both anti-oxidant carotenoids) is considered to involve increased GSH levels that protect against reactive oxygen species and apoptosis [23]. Similarly, crocin and crocetin may also suppress the activation of pro-inflammatory pathways (e.g., the nuclear factor kappa-B pathway) [23,50,51,52]. Furthermore, these two saffron constituents appear to enhance oxygen diffusion [53], and improve ocular blood flow [54], factors that play an important role in diseases such as AMD. Of note, pathways targeting not only oxidative stress and inflammation, but also endothelial function are also particularly relevant to glaucoma treatment and may mediate the effects of saffron and/or its constituents in patients with glaucoma. Indeed, the trabecular meshwork, which constitutes the target tissue of glaucoma in the anterior chamber, is comprised by endothelial-like cells, and is sensitive to oxidative damage, since it lacks effective antioxidant mechanisms [55,56]. As such, the trabecular meshwork is altered in most types of glaucoma, consequently increasing IOP [56], and may be a target for the effects of saffron and/or its constituents in patients with glaucoma; however, further research is required to clarify these potential mechanisms/effects. Finally, saffron and its constituents have been shown to improve glycaemia by enhancing insulin sensitivity and preventing pancreatic beta-cell failure [39,57], which in turn may improve diabetic retinopathy/maculopathy [39].

Moreover, a recent study by Corso et al. demonstrated that saffron can decrease ATP-induced retinal cytotoxicity by targeting the P2X7 receptors [58]. As these receptors are found in both inner and outer retinal cells, which are affected in neurodegenerative disorders such as AMD, this suggests another potential mechanistic pathway via which saffron may protect against neurodegeneration [58]. This is also supported by data showing that saffron may protect photoreceptors against retinal stress, maintaining both function and morphology in a mammalian retinal model after exposure to damaging bright continuous light [59]. Laabich et al. have also shown that crocin protects retinal photoreceptors against light-induced cell death in primary retinal cell cultures [60].

It should be also noted that the pharmacokinetic details regarding whether and which saffron constituents/metabolites reach the various tissues after oral intake of saffron or one of its constituents are not fully clarified yet. Therefore, the exact mechanisms by which saffron and/or its constituents act on the retina (particularly, on the photoreceptors and bipolar cells) remain to be further elucidated. Indeed, differences in uptake and tissue distribution may further account for different underlying pathways/mechanisms which can mediate the observed effects of oral saffron supplementation in patients with ocular disease. For example, lutein is considered to exert protective effects against AMD through its anti-oxidative properties and its accumulation in the macula acting as a blue light filter [61]. On the other hand, saffron and its constituents may improve ocular function more indirectly by improving the aforementioned systemic CVD-risk factors through anti-inflammatory and anti-oxidant effects [22,23,26,27,28,62]. 

Pharmacokinetic studies in rats have shown that after oral administration, crocin is largely excreted from the gastro-intestinal tract, which also serves as an important site for crocin hydrolysis [63]. As such, orally administered crocin is hydrolysed to crocetin before intestinal absorption [41,64]. Furthermore, crocetin has been shown to be quickly absorbed after oral administration in mice, with a short plasma half-life, resulting in rapid elimination without accumulation in the body [41]. Interestingly, the progress of saffron supplementation has been examined both in an animal model with light-induced retinal degeneration and AMD patients [42]. Indeed, experiments in albino rats under saffron treatment showed that crocetin was detectable in the collected blood samples [42]. However, there were no traces of saffron-related metabolites in any other examined tissue, with the exception of degenerating retinas where modest amounts of crocins were found in 7 of the 15 tested animals [42]. These findings suggest that crocins may be resynthesized from circulating crocetin, which can reach the retina following blood-brain barrier damage, as also supported by the fact that no crocin or crocetin metabolites were found in healthy retinas or other parts of the central nervous system [42]. In addition, based on analyses of blood and urine samples (collected within two hours after the morning saffron dose) from two AMD patients under saffron supplementation for over a year and three healthy volunteers after two weeks under the same saffron dose (20 mg daily), crocetin was consistently quantified in both blood and urine samples from the healthy volunteers, but not in those from the AMD patients, suggesting that these metabolites are immediately absorbed and used [42]. In line with this, data from an open-label, single dose escalation study (single crocetin dose of 7.5, 15, and 22.5 mg in one-week intervals) have also indicated that crocetin is absorbed more quickly than other carotenoids (e.g., lutein) in healthy adults [65]. 

Finally, an increasing body of RCTs indicate that saffron may improve the effects and symptoms of depression [47,66,67,68]. As depression may often affect patients with chronic diseases (e.g., with AMD, diabetic retinopathy, or glaucoma) [35,69,70] in whom it may also reduce treatment adherence (e.g., potentially lower adherence to glaucoma treatment, and, hence increase the glaucoma progression risk) [70], it seems plausible to hypothesize that some of the benefits of oral saffron supplementation in patients with ocular diseases may also have a component relating to the potential anti-depressive effects of saffron and/or its constituents [35]. However, currently there is a paucity of clinical data addressing this hypothesis that requires testing in the context of well-designed RCTs.

Overall, the majority of the data regarding the potential mechanisms/pathways mediating the effects of saffron and/or its constituents in ocular diseases originate from either *in vitro* experiments on human cell lines or animal models. These experiments/models allow the study of effects and mechanistic pathways that may be otherwise inaccessible, but have obvious limitations regarding their applicability to humans due to their nature (e.g., models involving light- or drug-induced ocular damage), which also cannot fully account for the multifactorial and progressive nature of most ocular diseases in humans. In addition, findings documented in clinical studies with saffron or its constituents that relate to subjective measures of vision and quality of life indices cannot be replicated and further tested in animal models. Thus, it becomes evident that translating and clarifying the evidence regarding the underlying mechanisms/pathways induced by saffron and/or its constituents in ocular diseases in humans requires additional data from both pre-clinical and clinical studies.

## 7. Summary of the Literature

The existing clinical evidence suggests that oral supplementation with saffron or crocin may have positive effects on various vision-related parameters in adults with AMD, POAG, and diabetic maculopathy (Table 2) [32,33,34,35,36,37,38,39]. Moreover, the findings from these clinical studies support the good safety profile of oral supplementation with saffron (range of tested daily doses: 20 to 50 mg) and crocin (5 mg and 15 mg daily) in these patients, but long term safety data are still scarce. As such, it is not possible to draw firm conclusions for evidence-based recommendations regarding oral saffron supplementation from the clinical studies conducted so far, since the existing data are considered rather limited, particularly regarding long term outcomes and for conditions other than AMD. In addition, further research is also required to clarify the exact underlying mechanisms that mediate the noted positive outcomes of oral saffron supplementation in ocular diseases. Based on the available data from *in vitro*, animal, and human studies, it is currently considered that pleiotropic effects of saffron and/or its constituents relating to anti-inflammatory and anti-oxidant pathways, as well as to improvements in oxygen diffusion and ocular blood flow are likely to facilitate the documented benefits of saffron supplementation in ocular diseases (Figure 1) [22,23,26,27,28,53,54,62]. 

## 8. Conclusions

Saffron supplementation appears to have promising potential as an effective and safe adjunct therapy in certain ocular diseases [32,33,34,35,36,37,38,39,61]. It is important to highlight that nutritional/dietary supplements (i.e., concentrated sources of vitamins and minerals and/or other substances with a nutritional or physiological effect that are marketed in “dose” form, such as tablets, capsules, pills, or liquids in measured doses) have to comply to certain national/international regulations [71,72]. Indeed, although such supplements are regulated as foods, these are subject to different regulations compared to other foods and from drugs in order to protect consumers against potential health risks from these products and the general public against potentially misleading information [72]. However, such regulations vary from country to country, despite certain shared existing regulatory frameworks (e.g., the European Directive on Food Supplements) [71,72]. Particularly for saffron, it is also noteworthy that its content may vary depending on the source [61]. In this context, it becomes evident that there is still an unmet need for additional bioavailability and pharmacokinetic studies, as well as well-designed, adequately powered and long term RCTs in order to form evidence-based recommendations for the potential therapeutic role of oral saffron supplementation in ocular diseases.

## Figures and Tables

**Table 1:** Predefined PICOS (Population/Participants/Problem, Intervention, Comparators, Outcomes, Study Design) criteria that were followed in order to identify and include clinical studies in the present review.

Parameters	Descriptions
**Population/Participants/Problem**	Adults with ocular disease
**Intervention**	Any intervention with oral administration of saffron or one of its constituents
**Comparison**	Studies with any comparator/control that incorporated a non-intervention group or studies with a pre- vs. post-intervention comparison without a comparator/control group
**Outcomes**	Vision-related outcome measures, such as visual acuity, visual field parameters, contrast sensitivity, electrophysiology parameters (ERG, fERG, mfERG), macular thickness measures, and IOP
**Setting**	Clinical studies/trials

ERG: electroretinography; fERG: focal electroretinography; mfERG: multifocal electroretinography; IOP: intraocular pressure.

**Table 2:** Clinical studies investigating the effects of oral supplementation of saffron or crocin on vision-related parameters in adults with ocular diseases.

Ocular Disease	Number of Subjects	Constituent Dosage	Study Design	Primary Outcome Measures—Findings	Proposed Mechanisms	Reference
AMD [bilateral early AMD]	*N* = 25	Saffron 20 mg daily	Double-blind, placebo controlled, cross over, RCT three-month period with cross over for another three months	fERG: increased amplitude in saffron, but not in placebo group BCVA: increased (one line) in saffron, but not in placebo group	Anti-oxidant Neuroprotective	Falsini et al. (2010) [32]
AMD [bilateral early AMD]	*N* = 29	Saffron 20 mg daily	Longitudinal interventional open-label study three monthly follow-ups over a 15-month period	fERG: increased amplitude that stabilized after three months BCVA: increased (two lines) that stabilized after three months	Anti-oxidant Neuroprotective	Piccardi et al. (2012) [33]
AMD [bilateral early AMD]	*N* = 33	Saffron 20 mg daily	Longitudinal, 3 monthly follow-ups over a 12-month period	fERG: increased amplitude and sensitivity amplitude that stabilized after three months independent of genotype	Anti-oxidant Anti-inflammatory Neuroprotective	Marangoni et al. (2013) [34]
AMD [dry and wet AMD]	*N* = 40	Saffron 15 mg twice daily	Placebo controlled, RCT six-month period with follow-ups at three and six months	CMT: decreased in saffron and placebo groups in wet AMD, but not in dry AMD ERG: amplitude increased in the saffron group (dry and wet AMD) compared to placebo after three months, but not six months	Neuroprotective Anti-depressant	Lashay et al. (2016) [35]
AMD [mild/moderate dry AMD]	*N* = 54	Saffron 50 mg daily	Placebo controlled, RCT three months	CMT: unchanged BCVA: increased (one line) in saffron, but not in placebo group CS: increased in saffron, but not in placebo group	Anti-oxidant Hemorheological activity	Riazi et al. (2017) [36]
AMD [mild/moderate AMD]	*N* = 96	Saffron 20 mg daily	Double-blind, placebo controlled, cross over, RCT three months followed by cross over into the other arm for three months	BCVA: increased in saffron group [and AREDS * + saffron], but not in placebo mfERG response density: increased in AREDS+saffron, but not in the saffron or placebo group mfERG latency: decreased in saffron group, but not in placebo group	Anti-oxidant Neuroprotective	Broadhead et al. (2019) [37]
POAG [clinically stable POAG]	*N* = 34	Saffron 30 mg daily	Double-blind, placebo controlled RCT 1 month duration 1 month wash-out	IOP: reduction after three and four weeks compared to placebo IOP returned to pre-intervention levels after a 4-week wash out period	Antioxidant Neuroprotective	Bonyadi et al. (2014) [38]
DME	*N* = 60 (101 DME eyes)	Crocin 5 mg or 15 mg daily	Double-masked, placebo controlled, phase 2 RCT 3 months	CMT: significantly decreased after three months compared to placebo only in the 15 mg group BCVA: significantly improved after three months compared to placebo only in the 15 mg group HbA1c and FBG: significantly decreased after three months compared to placebo only in the 15 mg group	Anti-oxidant Hemorheological activity Anti-inflammatory	Sepahi et al. (2018) [39]

*: participants were requested to continue on any supplements (including AREDS-based therapies) they had been taking prior to this study. AMD: age related macular degeneration; POAG: primary open angle glaucoma; DME: diabetic macular edema; RCT: randomized clinical trial; BCVA: best corrected visual acuity; AREDS: Age-Related Eye Disease Study; CMT: central macular thickness; CS: contrast sensitivity; ERG: electroretinography; fERG: focal electroretinography; mfERG: multifocal electroretinography; IOP: intraocular pressure; HbA1c: haemoglobin A1c; FBG: fasting blood glucose.
